# Religion’s Influence on Successful Aging: Mediating Mechanisms of Life Satisfaction and Depressive Symptoms Among Community-Dwelling Older Adults in Brazil

**DOI:** 10.1007/s10943-026-02605-6

**Published:** 2026-03-05

**Authors:** Leandro da Silva-Sauer, Rafael Sánchez-Vallejo, Gisele Cristina Resende, Bárbara Luque, Bernardino Fernández-Calvo

**Affiliations:** 1 Faculty of Psychology, Federal University of Amazonas, Manaus, Brazil; 2 Laboratory of Neuroscience, Neuropsychology, and Assessment, Federal University of Amazonas, Manaus, Brazil; 3 Department of Psychology, Faculty of Educational Sciences and Psychology, University of Córdoba, C/ San Alberto Magno S/N, 14071 Córdoba, Spain; 4 Applied Psychology Research Group, Maimonides Biomedical Research Institute of Cordoba, Córdoba, Spain; 5 Department of Psychology, Federal University of Paraíba, João Pessoa, Brazil

**Keywords:** Mental health, Intrinsic religiosity, Religious beliefs, Elderly, Subjective well-being

## Abstract

The global rise in the aging population highlights the need to understand factors contributing to successful aging. Intrinsic religiosity, defined as a deep, personal commitment to religious beliefs and practices, impacts well-being and mental health in older adults. This cross-sectional study investigates how life satisfaction and depressive symptoms mediate the relationship between intrinsic religiosity and successful aging. A sample of 538 Brazilian older adults (ages 60–101, M = 69.72, SD = 7.92; 58.6% female) completed the Satisfaction with Life Scale, the HADS Depression Scale, the Successful Aging Scale, and the Duke Religion Index. Sequential mediation analysis was conducted. Intrinsic religiosity significantly influenced successful aging indirectly through life satisfaction and depressive symptoms, with no significant direct effect. These findings suggest that intrinsic religiosity may enhance the successful aging of older adults by improving life satisfaction and reducing depressive symptoms. Integrating these insights into social policies, that is, supporting older adults’ intrinsic religiosity, might help many Brazilian older adults achieve successful aging.

## Introduction

Healthy aging demands a comprehensive understanding of the biological, psychological, and social factors that sustain well-being in later life. The successful aging framework proposed by Rowe and Kahn, (1997) defines this construct through three criteria: low risk of disease and disability, high cognitive and physical functioning, and active engagement with life. Although the construct has continued to evolve (Cosco, 2014), important gaps persist in the literature, particularly in middle-income countries, concerning the demographic consequences and intergenerational distribution of aging-related costs (Orgura & Jakovleivic, 2018). Furthermore, the role of socio-cultural determinants, specifically religion, remains under-examined, necessitating research that grounds successful aging within specific cultural landscapes (Estebsari et al., 2020).

Recent evidence reinforces the centrality of religiosity across aging populations worldwide. In Brazil, the interplay between resilience, physical activity, and religiosity promotes better mental health outcomes (da Silva-Sauer et al., 2025). Similarly, longitudinal data from Thailand links religious involvement to enhanced mental and behavioral health (Pengpid & Peltzer, 2025), while North American studies indicate that institutional religious participation serves as a robust predictor of longevity (Brown et al., 2025). Brazil provides a particularly compelling context for this study: 95% of the population identifies with a religion and 83% considers faith highly important, making religiosity a structural, not merely personal, resource that offers social, emotional, and spiritual support to older adults (Abdala et al., 2015; Moreira-Almeida et al., 2010).

Religiosity predicts positive aging outcomes that extend beyond physical health (Crowther et al., 2002; Papadopoulos, 2020), with evidence indicating a moderate positive relationship between religious involvement and successful aging (da Silva-Sauer et al., 2022). This association is not confined to organized religion; spirituality and personal meaning-making outside formal institutional practice contribute comparably (Koenig et al., 2012). Religious engagement supports successful aging through multiple pathways: it facilitates stress coping, improves health outcomes, enhances cognitive functioning, expands social networks, and attenuates depressive symptoms by reinforcing emotional coping and sustaining broader dimensions of well-being (Emmons & Paloutzian, 2003; Koenig et al., 1997; Lee & Zhang, 2018; Moberg, 2008; Salamene et al., 2021; Silberman, 2005; Tavares, 2019).

Among the dimensions of religiosity, intrinsic religiosity holds particular theoretical relevance: it denotes an orientation in which religion is not a means to external ends but an ultimate end in itself, guiding values and everyday conduct (Koenig & Büssing, 2010). This construct traces its origins to Allport’s foundational distinction between intrinsic and extrinsic religious orientations (Masters, 2013), and identifies individuals for whom faith operates as the organizing principle of daily life.

Life satisfaction represents a critical pathway through which religiosity may influence successful aging (Bhattacharyya et al., 2024; Depp & Jeste, 2006). Defined as an individual’s global positive evaluation of life (Diener, 2000), it is inversely associated with depression and positively linked to favorable aging outcomes (da Silva Sauer et al., 2021; Van Damme Ostapowicz, 2021). Religiosity enhances life satisfaction across cultural settings by fostering positive emotions (Foong et al., 2020; J. Park et al., 2012; Yoon et al., 2004). In turn, high life satisfaction encourages healthier lifestyles, stronger social engagement, and better cognitive and physical functioning (Bhattacharyya et al., 2022; Chu & Koo, 2023), while buffering depressive symptoms, particularly in the presence of an optimistic disposition (Bhattacharyya et al., 2024; Chen et al., 2020). Understanding its mediating role is therefore essential for designing interventions that integrate the subjective and objective dimensions of successful aging.

Depression, by contrast, represents an opposing pathway that systematically undermines successful aging (da Silva-Sauer et al., 2020; Jeste et al., 2013; Vahia et al., 2010) by impairing physical activity, nutrition, social participation, and future orientation (Golja et al., 2020; Salazar-Barajas et al., 2018). Even subsyndromal symptoms shape aging outcomes through biological pathways, such as inflammation and chronic stress, that accelerate cognitive decline (O’Hara, 2006). The relationship is bidirectional: reduced social engagement aggravates depressive symptoms, which in turn further limits engagement (Taylor & Lynch, 2004). Addressing depression is therefore as consequential for successful aging as alleviating physical disability (Jeste et al., 2013; Vahia et al., 2010). Given the protective role of religiosity against depression and the centrality of both constructs to successful aging, understanding their sequential relationship through life satisfaction becomes theoretically essential.

The present study builds on this theoretical framework to examine the mechanisms through which intrinsic religiosity shapes successful aging. We hypothesize that intrinsic religiosity influences successful aging indirectly, operating through enhanced life satisfaction and, subsequently, reduced depressive symptoms. Specifically, we test a serial multiple mediation model in which life satisfaction and depressive symptoms function as sequential mediators: intrinsic religiosity enhances life satisfaction, which in turn reduces depressive symptoms, ultimately contributing to successful aging. By specifying these psychological pathways, the study treats successful aging as a biopsychosocial construct rather than a clinical outcome alone. This approach allows us to capture the interplay between subjective well-being, mental health, and religious experience in later life. The findings provide an empirical basis for interventions that leverage religious and community-based resources to improve the quality of life of older adults, with implications for both clinical practice and public health policy in aging populations.

## Method

### Participants and Ethics

The sample consisted of 538 Brazilian older adults, including 315 Female (58.6%), with ages ranging from 60 to 101 years (M = 69.72, SD = 7.92). All resided in the northeastern states of Paraíba, Pernambuco, and Rio Grande do Norte, were native Portuguese speakers, and demonstrated sufficient independence to complete the study instruments without severe sensory or physical impairments.

Inclusion criteria required participants to be (1) 60 years or older, following World Health Organization criteria for older adults in developing countries, and (2) cognitively able to understand and respond to questions in written (literate participants) or oral form (low literacy), with no dementia history.

The study complied with Resolution 196/96 of the Brazilian National Health Council and the Helsinki Declaration. The institutional research ethics committee approved the protocol (no. 10711113.4.0000.5188). All participants provided written informed consent. Participation was voluntary and non-remunerated.

### Measures

Sociodemographic information included sex (male/female), years of education, self-declared skin color (yellow, white, indigenous, black, or brown), and marital status (single, married, widowed, divorced/separated, other).

Religious involvement was measured with the Duke University Religion Index (DUREL; Koenig & Büssing, 2010), validated in Brazil by Lucchetti et al., (2012). The instrument comprises five items assessing organized religious activities, non-organized religious activities, and intrinsic religiosity. Only the intrinsic religiosity subscale (DUREL-IR) was used. This three-item measure (e.g., “I feel the presence of God in my life”), uses a 5-point scale (1 = “Not true” to 5 = “Totally true for me”), yielding scores from 3 to 15. Higher scores indicate stronger intrinsic religious orientation. Prior studies have linked DUREL-IR to psychological well-being and reduced depressive symptoms in older adults (Laurencelle et al., 2002; Nelson, 1990).

Life satisfaction was assessed with the Satisfaction with Life Scale (SWLS; Diener et al., 1985), adapted for Brazil by Neto (1993). The SWLS consists of five items (e.g., “In most ways, my life is close to my ideal”), rated on a 7-point Likert scale (1 = “Strongly disagree” to 7 = “Strongly agree”), with total scores ranging from 5 to 35. Higher scores reflect greater life satisfaction.

Depressive symptoms were measured using the depression subscale of the Hospital Anxiety and Depression Scale (HADS-D; Snaith & Zigmond, 1986), validated in Brazil by Botega et al., (1995). Seven items (e.g., “I feel slowed down”) are rated from 0 to 3, yielding scores from 0 to 21. Higher scores indicate more severe depressive symptoms.

Successful aging was evaluated with the Successful Aging Scale (SAS; Reker, 2009), adapted for Brazil by da Silva-Sauer et al., (2020). The 13-item Brazilian version measures three dimensions: Healthy Lifestyle, Adaptive Coping, and Life Engagement. Responses use a 7-point Likert scale (1 = “Strongly agree to 7 = “Strongly disagree”), with total scores ranging from 13 to 91. Only the total score was analyzed as a global indicator of successful aging from a biopsychosocial perspective. Higher scores reflect more positive perceptions of aging and better adaptation to later life.

### Design and Procedure

This cross-sectional study employed a quantitative approach within an exploratory serial mediation framework involving data collection through structured questionnaires. As the study aimed to explore potential pathways linking religiosity, life satisfaction, depressive symptoms and successful ageing, mediation analysis was considered the most appropriate statistical strategy. This approach enables the testing of theoretical assumptions regarding indirect and sequential effects, rather than focusing solely on bivariate associations.

Participants were recruited through nonprobability convenience sampling across three northeastern Brazilian states (Paraíba, Pernambuco, and Rio Grande do Norte) from neighborhood associations, dental offices, community centers, beaches, subway stations, educational institutions, occupational therapy offices, churches, and commercial establishments. All individuals who met inclusion criteria and received study information agreed to participate; no refusals were recorded.

Licensed psychologists with research experience collected data face-to-face following standardized training and a written protocol. Supervision occurred throughout fieldwork. Questionnaires were administered in fixed order: sociodemographic questionnaire, DUREL-IR, SWLS, HADS-D, and SAS. For participants with low literacy, items were read aloud to ensure comprehension. Sessions lasted approximately 7 min.

### Data Analysis

Descriptive statistics and Pearson correlations examined bivariate associations among study variables. Pearson’s test was appropriate given approximate normal distributions, confirmed by skewness and kurtosis values within recommended ranges (Kline, 2023).

To test the hypothesized indirect and sequential effects, a serial mediation analysis was conducted using PROCESS macro for IBM SPSS (Model 6; Hayes, 2013, 2018). The model assessed whether the relationship between intrinsic religiosity (independent variable) and successful aging (dependent variable) was mediated by life satisfaction (Mediator 1) and depressive symptoms (Mediator 2). Three indirect pathways were tested: (*path* a) via life satisfaction, (*path* b) via depressive symptoms, and (*path* c) sequentially via both mediators. This analytic strategy was selected because it enables a rigorous and transparent assessment of indirect and sequential pathways, moving beyond simple bivariate associations.

Indirect effects were estimated using 10,000 bootstrap samples with bias-corrected 95% confidence intervals. Sociodemographic variables were tested as covariates, but their inclusion did not alter the significance or pattern of the results; therefore, the final models were estimated without them for parsimony. Assumptions of linearity, independence, homoscedasticity, and absence of multicollinearity were examined and met.

All analyses were conducted using IBM SPSS v27.0 (SPSS Inc., Chicago, IL, USA), with statistical significance set at p < .05.

## Results

Table 1 presents the descriptive characteristics of the sample (N = 538), which included a higher proportion of Female. Mean age was 69.72 years (SD = 7.92, range 60–101). Overall, participants reported moderate to high levels of intrinsic religiosity, life satisfaction, and successful aging, with low depressive symptoms. The sample was predominantly White or Brown, with most participants married or widowed.

**Table 1:** Descriptive statistics of the sample

Variables	Frequency (%)	DUREL-IR M (SD)	SWLS M (SD)	HADS-D M (SD)	SAS M (SD)
*Sex*					
Male	41.4	12.8 (2.8)	26.9 (5.5)	5.4 (3.9)	74.2 (9.9)
Female	58.6	13.4 (2.4)	25.9 (6.1)	5.2 (3.5)	75.2 (9.2)
*Skin color selfdeclared*					
Yellon	3	13.75 (3.0)	29.6 (3.1)	3.8 (2.7)	73.3 (9.9)
White	47.2	13.26 (2.4)	26.3 (6.4)	5.1 (4.0)	76.8 (9.7)
Indian	0.4	15.00 (0.0)	24 .0(0)	7.0 (0)	78.0 (0)
Black	6.7	13.36 (2.2)	26.3 (4.7)	6.4 (3.5)	70.6 (8.8)
Brown	42.8	13.09 (2.5)	26.2 (5.6)	5.4 (3.5)	72.9 (8.9)
*Marital status*					
Single	7.8	13.54 (2.7)	27.4 (3.7)	5.0 (3.3)	75.2 (7.3)
Married	52.8	13.15 (2.5)	27.3 (5.8)	5.0 (3.6)	76.0 (9.1)
Widowed	21.9	13.10 (2.5)	25.2 (6.4)	6.3 (3.9)	72.2 (9.7)
Divorced/separated	15.2	13.40 (2.3)	24.4 (6.1)	5.0 (4.1)	73.2 (11)
Other	2.2	13.58 (2.1)	25.3 (5.2)	4.1 (1.1)	74.5 (9.7)

N = 538; DUREL-IR = Duke University Religion Index, intrinsic religion dimension; SWLS = Satisfaction with Life Scale; HADS-D = Hospital Anxiety and Depression Scale, depression subscale; SAS = Successful Aging Scale; M = Mean; SD = standard deviation

### Bivariate Correlations

As shown in Table 2, intrinsic religiosity was positively correlated with life satisfaction and successful aging, and negatively correlated with depressive symptoms. Life satisfaction and depressive symptoms showed a strong inverse correlation, underscoring their interconnection. Life satisfaction was positively associated with successful aging, while depressive symptoms were negatively associated.

**Table 2:** Correlation matrix of study variables

Variable	DUREL-IR	SWLS	HADS-D	SAS	M (SD)	α
DUREL-IR	–				13.21 (2.5)	.79
SWLS	.318^**^	–			26.38 (5.9)	.84
HADS-D	−.283^**^	−.550^**^	–		5.32 (3.8)	.77
SAS	.242^**^	.440^**^	−.534^**^	–	74.69 (9.6)	.80

N = 538; DUREL-IR = Duke University Religion Index, intrinsic religion subscale; SWLS = Satisfaction with Life Scale; HADS-D = Hospital Anxiety and Depression Scale, depression subscale; SAS = Successful Aging Scale; M = Mean; SD = standard deviation; α: Cronbach’s alpha coefficient for each scale. * *p* < .05, ** *p* < .01

### Sequential Mediation Model

**Fig. 1:**
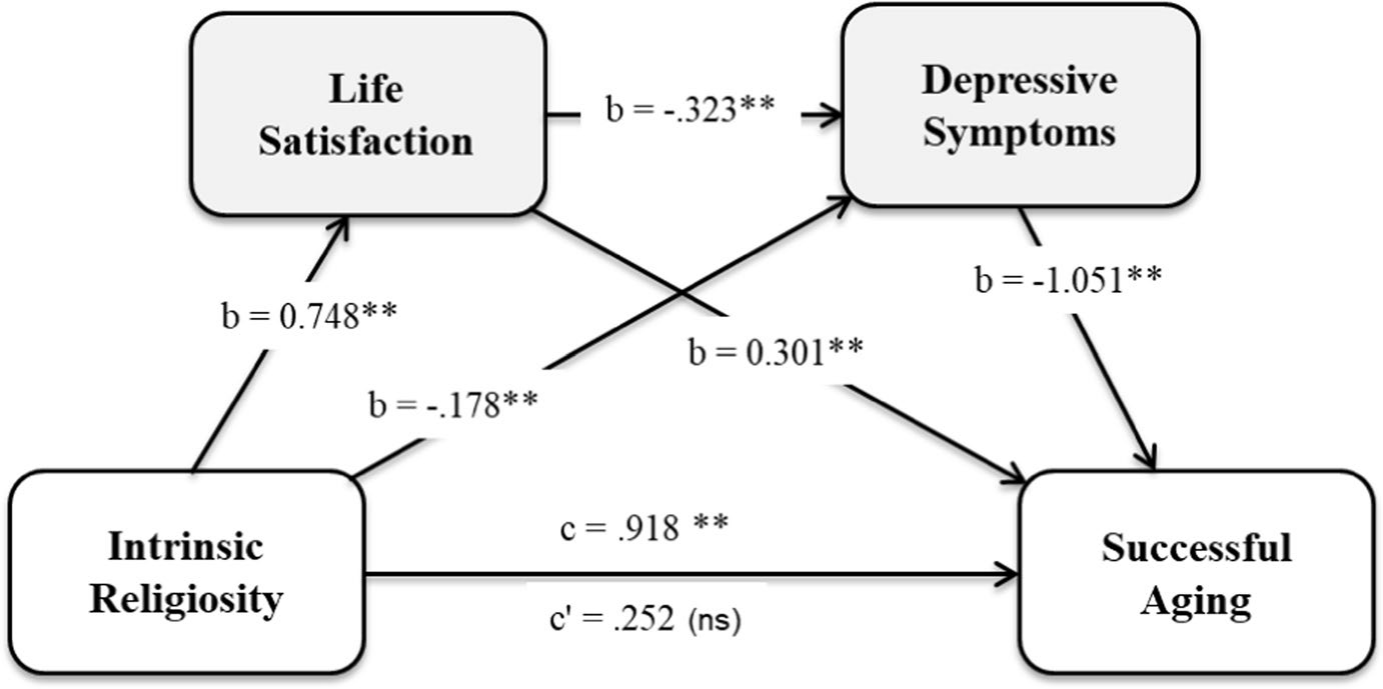
Serial Mediation Model of the Relationship Between Intrinsic Religiosity and Successful Aging. Serial mediation model examining the relationship between intrinsic religiosity and successful aging through life satisfaction (Mediator 1) and depressive symptoms (Mediator 2). Path coefficients represent unstandardized regression coefficients (b). The model tested three indirect pathways: (1) intrinsic religiosity → life satisfaction → depressive symptoms → successful aging (sequential mediation), (2) intrinsic religiosity → life satisfaction → successful aging, and (3) intrinsic religiosity → depressive symptoms → successful aging. The total effect was significant (c = .918, *p* < .001), while the direct effect was not significant after controlling for both mediators (c' = .252, *p* = .081), indicating full mediation. The model accounted for 31.8% of the variance in successful aging (*R*.^2^ = .318). *N* = 538. *p* < .05. *p < .01

Figure 1 displays the serial mediation model results. The total effect of intrinsic religiosity on successful aging was significant (c = .918, SE = .159, t = 5.78, *p* < .001). Three indirect pathways were tested. The indirect effect through life satisfaction alone was significant (.225, BootSE = .069, 95% BCa CI [.106, .374]). The indirect effect through depressive symptoms alone was also significant (.187, BootSE = .070, 95% BCa CI [.058, .335]). The sequential indirect effect through both mediators was significant (.254, BootSE = .057, 95% BCa CI [.151, .373]). The direct effect of intrinsic religiosity on successful aging, controlling for both mediators, was not significant (c' = .252, SE = .144, t = 1.75, *p* = .081). These results indicate that the relationship between intrinsic religiosity and successful aging operates indirectly through enhanced life satisfaction and reduced depressive symptoms. The sequential pathway, in which intrinsic religiosity enhances life satisfaction, which in turn reduces depressive symptoms, accounted for the largest indirect effect. Overall, the model accounted for 31.8% of the variance in successful aging (R^2^ = .318, F(3, 530) = 82.47, p < .001).

## Discussion

The primary objective of this study was to examine whether life satisfaction and depressive symptoms mediate the relationship between intrinsic religiosity and successful aging in Brazilian older adults. The findings support this hypothesis: intrinsic religiosity does not exert a significant direct effect on successful aging after controlling for mediators, but operates indirectly through enhanced life satisfaction and reduced depressive symptoms. This sequential mediation model clarifies mechanisms underlying previous evidence linking religiosity to positive aging outcomes (Carver & Buchanan, 2016; Lee, 2007; Lee & Zhang, 2018; Salamene et al., 2021), demonstrating that the relationship is not linear but mediated by psychological processes.

The first mediating pathway (life satisfaction) suggests that individuals who regard religious faith as central to their identity report greater life satisfaction. This aligns with evidence that religiosity provides emotional support, meaning, and a framework for coping with adversity (Bastos et al., 2023; Krause, 2003; Schieman et al., 2005; Villani et al., 2019). Religiosity also fosters a sense of *mattering*, the perception that one’s life has value and importance, which is associated with reduced loneliness, stronger social connections, and greater self-esteem (Schieman et al., 2010; J. Taylor & Turner, 2001). Within this framework, intrinsic religiosity strengthens resilience by promoting purpose and psychological support (Koenig et al., 2001).

The second pathway (depressive symptoms) demonstrates that religiosity is associated with lower depressive symptoms, consistent with studies highlighting its role in promoting adaptive coping mechanisms and emotional regulation strategies Emmons & Paloutzian, 2003; Lee & Zhang, 2018; Tavares, 2019). Specifically, intrinsic religiosity has been linked to more adaptive emotional regulation strategies (Mosqueiro et al., 2015; Vishkin et al., 2016), which function as resilience resources with protective effects on mental health in older adults (da Silva-Sauer et al., 2021; Moreno-Agostino et al., 2022). Some studies indicate that spirituality may influence mental health and life satisfaction as strongly as, or more strongly than, resilience itself (Shabani et al., 2023), underscoring the role of religiosity in reinforcing adaptive resources that support psychological well-being (Krause, 2003; Manning, 2014) and positive aging outcomes (Papadopoulos, 2020).

The sequential pathway, wherein intrinsic religiosity enhances life satisfaction, which in turn reduces depressive symptoms, revealed the strongest indirect effect. This suggests that religiosity contributes most effectively to successful aging when it simultaneously enhances satisfaction and mitigates depression. These mechanisms align with evidence that individuals with strong intrinsic religiosity view faith as a motivating force for meaningful engagement in daily activities (Byrd et al., 2007). Religious participation is associated with reduced health-risk behaviors (Pule et al., 2019), greater subjective well-being (Bastos et al., 2023), and a stronger purpose in life (You & Lim, 2019). These processes may protect against depression and foster better physical and cognitive functioning (Sutin et al., 2022), supporting adaptation, quality of life, and ultimately successful aging.

### Strengths and Limitations

This study has several strengths. The large and diverse sample of Brazilian older adults, recruited across multiple community contexts, increases ecological validity. Validated measures and a theoretically grounded mediation model enabled nuanced analysis of indirect effects. Rigorous data collection protocols, quality control procedures, and robust statistical analyses strengthen the credibility of the findings.

Limitations should also be acknowledged. First, the cross-sectional design precludes causal inferences and assessment of changes over time. Longitudinal designs are needed to confirm the directionality of effects. Second, the high prevalence of religiosity in Brazil (Romero, 2020) limits generalizability to less religious societies. Finally, reliance on self-report measures of intrinsic religiosity and successful aging may overlook objective health indicators or behavioral dimensions of religious practice. Future studies incorporating longitudinal designs, diverse cultural contexts, and both subjective and objective measures could clarify these relationships and strengthen causal inference.

### Purpose and Implications

Despite these limitations, this study provides novel evidence from a middle-income country, addressing a gap in the literature that has focused predominantly on high-income nations. By identifying life satisfaction and depressive symptoms as mediators, the findings clarify how religiosity contributes to successful aging. These results have direct implications for interventions aimed at promoting successful aging. Programs that integrate strategies to enhance life satisfaction and reduce depressive symptoms, potentially in collaboration with religious or community-based organizations, may be particularly effective in supporting successful aging. At the same time, interventions should recognize that non-religious sources of purpose and social connection can serve similar functions, ensuring inclusivity across diverse belief systems. These findings inform the development of public health policies that acknowledge religious engagement as one of several meaningful pathways to successful aging.

## Conclusions

This study demonstrates that the relationship between intrinsic religiosity and successful aging in Brazilian older adults is fully mediated by life satisfaction and depressive symptoms. By providing empirical evidence from a middle-income country, the findings broaden the cultural scope of research on aging and religiosity, underscoring the importance of psychosocial mechanisms in successful aging. Future research using longitudinal and cross-cultural designs, and incorporating objective indicators of health and functioning, will be essential to further clarify these pathways and strengthen evidence for interventions promoting successful aging across diverse populations.
